# Update on the Value of Lung Ultrasound Examination in Acute Decompensated Heart Failure Patients with Various Left Ventricular Ejection Fraction

**DOI:** 10.31083/j.rcm2310350

**Published:** 2022-10-18

**Authors:** Hui Zhang, Yunlong Zhu, Na Li, Jianping Zeng

**Affiliations:** ^1^Department of Cardiology, Xiangtan Central Hospital, 411100 Xiangtan, Hunan, China; ^2^Graduate Collaborative Training Base of Xiangtan Central Hospital, Hengyang Medical School, University of South China, 421001 Hengyang, Hunan, China

**Keywords:** pulmonary congestion, lung ultrasound, ADHF, HFrEF, HFmrEF, HFpEF

## Abstract

Acute decompensated heart failure (ADHF) is one of the most common causes of hospital admission for cardiovascular diseases. ADHF often affects the elderly population, is associated with high morbidity, admission rate and mortality. Pulmonary congestion (PC) is the most common cause of hospitalization among ADHF patients. Previous studies have shown that lung ultrasound (LUS) serves as a valuable tool for the evaluation of PC in patients with heart failure in terms of diagnosis, guiding of the treatment, and post-discharge monitoring. The use of LUS for ADHF is well described and already widely used in the daily clinical practice. PC might differ in ADHF patients with different left ventricular ejection fraction value and treatment options should be steadily adjusted according to the LUS-derived PC results to improve the outcome. This review summarized the value of LUS examination in patients with ADHF with preserved, mildly reduced, and reduced left ventricular ejection fraction, aiming to expand the rational use of LUS, promote the LUS-guided management and improve the outcome among patients with ADHF.

## 1. Introduction

Heart failure (HF) is a global public health problem affecting 26 million people worldwide [1]. Acute decompensated heart failure (ADHF) refers to the acute attack of symptoms and signs of HF [2]. The common causes of ADHF include drug and dietary disorders, arrhythmia, deterioration of renal function, poor control of hypertension, myocardial infarction, and infection [3]. Risk of readmission within 6 months was as high as 50% for ADHF patients hospitalized due to worsening HF [4], and repeated hospitalization will seriously affect the quality of life of ADHF patients. The prognosis of ADHF patients is also poor. After discharge, the 1-year mortality rate is around 40–45% [5, 6], and the 5-year mortality rate is about 70–80% [7, 8] among ADHF patients.

In the 2016 European Society of Cardiology (ESC) guidelines for the diagnosis and treatment of acute and chronic heart failure, HF patients were classified according to left ventricular ejection fraction (LVEF) [9]. Patients were defined as heart failure with reduced ejection fraction (HFrEF) (LVEF <40%), heart failure with mid-range ejection fraction (HFmrEF) (LVEF 40%–49%), and heart failure with preserved ejection fraction (HFpEF) (LVEF ≥50%) [9]. In the 2021 ESC guidelines for the diagnosis and treatment of acute and chronic heart failure, HFmrEF was renamed as heart failure with mildly reduced ejection fraction (LVEF 41%–49%) [10]. A study on the prognosis of de novo acute heart failure (AHF) and ADHF showed that HFrEF accounted for 60.8%, HFmrEF for 15.1%, and HFpEF for 24.1% respectively of ADHF patients [11]. Onteddu *et al*. [12] reported that HFrEF accounted for 77%, HFmrEF for 11%, and HFpEF for 12%, respectively of ADHF patients. Jayagopal *et al*. [13] showed that HFrEF accounted for 72.4% and HFmrEF and HFpEF accounted for the rest 27.6% of ADHF patients. HFrEF is thus the largest patient group of ADHF. Since HFpEF patients accounted for the main population in the overall HF patients [14], patients with HFrEF are thus more prone to cardiac decompensation than patients with HFmrEF and HFpEF.

HF is the final stage of various heart diseases. Pulmonary congestion (PC) is a common sign of HF and directly related to the major HF symptom (dyspnea) and signs of HF (pulmonary rales, peripheral edema, and vascular congestion) [10]. In case of ADHF, pulmonary edema and the rapid accumulation of fluid within the interstitial and alveolar spaces could lead to more significant dyspnea and respiratory decompensation. The causes of pulmonary edema are multiple, cardiogenic pulmonary edema is usually a result of acutely elevated cardiac filling pressures [15]. Cardiogenic pulmonary edema refers to hemodynamic PC with increased capillary pressures. This could result in increased fluid transfer out of capillaries into the interstitium and alveolar spaces. High capillary pressures can further cause barrier disruption, which increases the permeability and fluid transfer into the interstitium and alveoli. Fluid in alveoli could alter surfactant function and increases surface tension. This vicious circle can lead to more edema formation and to atelectasis with impaired gas exchange [16]. PC is thus the most common feature in patients with ADHF.

## 2. The Use of Lung Ultrasound for Acute HF in Emergency Department

The use of LUS for ADHF is well described [17, 18] and already widely used in the daily clinical practice, especially in emergency department (ED). The emergency department is very important for the diagnosis and treatment of patients with acute dyspnea, and the important etiology of dyspnea is ADHF [19]. The use of LUS images is helpful for the diagnosis of patients with acute respiratory failure, circulatory shock, or cardiac arrest. LUS scores can be used to quantify lung ventilation and thus regulate the parameters of ventilators in mechanically ventilated patients, LUS can also be used for early detection and management of respiratory complications under mechanical ventilation, such as pneumothorax, ventilator-associated pneumonia, atelectasis, and pleural effusions [20]. In the ED, there are two regimens for the use of LUS. One is the bedside lung ultrasound in emergency (BLUE)-protocol, which is primarily used to rapidly diagnose the cause of acute respiratory failure. The other one is the fluid administration limited by lung sonography (FALLS)-protocol, which is used to guide the management of acute circulatory failure [21]. The BLUE protocol is used in the diagnosis of patients with ADHF, which required scanning 12-point of the chest, and 8-point or 6-point is sufficient to quickly diagnose AHF [22, 23]. In critically ill patients, 4-point or 2-point can be used to identify lung conditions. Sforza A *et al*. [24] found that bilateral chest LUS was more sensitive and accurate to the diagnosis of AHF than double anterior chest LUS, and with the exacerbation of hypoxemia, the accuracy of anterior chest LUS in the diagnosis of AHF gradually increased and approached that of lateral chest LUS. In the first 6 hours of ED management, there was no significant difference in the degree of PC response caused by LUS-guided treatment compared with usual care in patients with ADHF, but LUS-guided treatment improved PC more quickly within 48 hours [25]. Therefore, repeating LUS examination at 6 hours after admission is meaningful, which could be helpful for the medication adjustment, and for the improvement of the PC status within 48 hours after admission for ADHF patients.

In ED, LUS can quickly guide the diagnosis and treatment of acute respiratory distress syndrome (ARDS), COVID-19 pneumonia, interstitial lung disease, and other diseases [26, 27, 28]. For patients with HF, as long as the patient has signs of PC, whether it is AHF or CHF, HFrEF, HFmrEF, or HFpEF, LUS is an important tool for diagnosis and treatment. Unlike echocardiography, LUS does not require professional operation skills, as long as it avoids the ribs, it can be examined in the whole chest. All ultrasound equipment suitable for the abdomen and superficial organs can meet the requirements of lung ultrasound. Convex array, phased array, and linear array probes can be used for a LUS examination. High frequency linear array probe (7.5~10 MHz) is mainly used for the examination of the chest wall, pleura and sub-pleural lesions. Low frequency convex array probe (2~5 MHz) is suitable for the examination of deep lung tissue lesions and obese people. All these provide convenience for the clinical application of LUS.

## 3. Clinical Value of Lung Ultrasound Examination in the Diagnosis, Treatment and Monitoring of Acute Decompensated Heart Failure Patients

Lung ultrasound (LUS) examination could sufficiently evaluate PC through detecting the presence and number of B-lines, which indicate pulmonary edema or loss of lung aeration [29]. The advantage of B-line assessment is that it could provide direct information on pulmonary interstitial lesions and can distinguish between hemodynamic congestion and pulmonary interstitial edema. The signs of PC on LUS are often shown as B-line evenly distributed on both sides with smooth pleural line, the regularly spaced B-line shows septal or interstitial edema, while the crowded or combined B-line shows alveolar edema. ADHF patients can sometimes present in the form of many diffused B-lines with strong echo in the whole lung field, at this time, it is called “white lung”. LUS can easily detect pulmonary edema in patients with acute decompensation and in patients at risk for decompensation. LUS could also help characterize patients with cardiogenic pulmonary edema and help identify subgroups who need specific management [30, 31, 32]. As a useful tool for evaluating PC, LUS could significantly contribute to the diagnosis of ADHF (Table 1, Ref. [33, 34, 35]) and has become standard care in many emergency departments and intensive care settings [36].

**Table 1.** **Study on the diagnostic value of LUS in ADHF patients**.

Study	N	Conclusions
Pivetta *et al*. (2015) [33]	1005	LUS combined with clinical examination can improve the diagnosis of ADHF.
Pivetta *et al*. (2019) [34]	518	Integration of LUS with clinical assessment for the diagnosis of ADHF in the emergency department seems to be more accurate than the current diagnostic approach based on CXR and NT-proBNP.
Hacıalioğulları *et al*. (2021) [35]	80	In the ED setting, an assessment of B-lines and measurement of IVC diameters are better markers than the B-type natriuretic peptide level, EF, or chest x-ray for diagnosis of ADHF and can be used to make decisions for hospitalization or discharge from the ED.

Abbreviations: LUS, lung ultrasound; ADHF, acute decompensated heart failure; CXR, chest radiography; NT-proBNP, N-terminal pro-B-type natriuretic peptide; ED, emergency department; IVC, inferior vena cava; EF, ejection fraction.

LUS is helpful for the early and rapid diagnosis of ADHF and can improve the treatment efficacy for these patients [37]. The most important scheme to reduce PC in clinical practice is the proper use of diuretics. A prospective clinical trial has shown that early intravenous diuretics could reduce in-hospital mortality, while in-hospital mortality increased by 2.1% for each 4-hour delay in the use of intravenous diuretics [38]. BLUSHED-AHF is a multicenter, single-blind prospective clinical study, 130 patients with AHF admitted in the emergency department were enrolled and divided into LUS guided nursing group and routine nursing group at 1:1 ratio within the first 6 hours of treatment [25]. Within 48 hours, the remission of PC was faster in the LUS guided nursing group than that in the routine nursing group [25]. Studies have shown that the risk of adverse events and long-term adverse prognosis in patients with HF increases in proportion with the increased number of B-lines [39]. LUS is thus a sensitive tool for risk stratification of ADHF [39]. It can be added to the remote monitoring item of patients with HF and serves as an important tool of telemedicine. Another study clearly showed that timely management of patients at risk of decompensation based on LUS results can reduce the risk of HF deterioration and prevent readmission [40]. Since PC and outcome might differ among ADHF with various LVEF, it is of importance to define PC status in ADHF patients with various LVEF to develop LVEF- and PC-oriented monitoring and therapy strategies for these patients.

## 4. Pulmonary Congestion in Acute Decompensated Heart Failure Patients with Reduced Ejection Fraction

HFrEF refers to HF patients with LVEF ≤40%. HFrEF is often initially characterized by decreased cardiac output, which further leads to a series of adverse reactions [41]. Hemodynamic features of left ventricular dysfunction in ADHF with HFrEF include: the temporary effect of deteriorated left ventricle systolic function; subsequent compensation by activating the sympathetic nervous system and renin-angiotensin-aldosterone system (RAAS) [42]. When the heart is severely damaged and decompensated, RAAS activation could not maintain cardiac output to meet the oxygenation needs of vital organs and peripheral circulation. PC, dyspnea and fluid retention form the usual clinical symptoms and signs of HFrEF patients [43]. The reduced cardiac output could lead to progressive retention of blood volume fluid. Progressive increase of systemic filling pressure and right atrial pressure, increased LV end diastolic pressure (LVEDP), which could collectively contribute to the formation of PC. Thus, detecting and monitoring PC plays a central role in the management of ADHF patients with reduced EF.

Miglioranza *et al*. [44] evaluated the association between PC and decompensation HF patients with LVEF <45%, they found that in the outpatient department of HF, the degree of PC, assessed by LUS, was significantly correlated with decompensation. B-lines ≥15 can be considered as a quick and reliable biomarker of decompensation in outpatients with heart failure and reduced LVEF. A study evaluated the association between PC incidence and short-term and long-term prognosis in patients with AHF and reduced LVEF, results showed that there was no significant difference in PC among AHF patients with chronic obstructive pulmonary disease (COPD) and other comorbidities and different EF, while there was a stronger association between the degree of PC and early events after discharge (*p* = 0.022) [44]. Scali *et al*. [45] evaluated the effect of exercise-induced PC on prognosis in patients with HFrEF and found that twelve-month event-free survival was 95% in the 36 patients with stress B-lines <30 (best cut-off identified by receiver operating characteristic curve analysis) versus 7% in patients with ≥30 B-lines (*p *< 0.0001). Bajraktari *et al*. [46] verified that LUS and B-type natriuretic peptide (BNP) guided follow-up care can improve the survival rate of patients with HFrEF.

## 5. Pulmonary Congestion in Acute Decompensated Heart Failure Patients with Mildly Reduced Ejection Fraction

HFmrEF is defined as a clinical syndrome with an EF of 41%–49%, typical HF symptoms and signs in patients with structural heart disease. In all patients with heart failure, the prevalence rate of HFmrEF is around 10%–20% [47]. Compared with the population of HFrEF and HFpEF, patients with HFmrEF showed general clinical characteristics between HFpEF and HFrEF [47]. Patients with HFmrEF can be further divided into three subgroups: HFmrEF improved (previous LVEF <40%), HFmrEF deteriorated (previous LVEF >50%), HFmrEF remained unchanged (previous LVEF was 41%–49%).

Although the concept of HFmrEF has been proposed for several years, and the research on HFmrEF is far less than HFrEF and HFpEF. At present, there are relatively few studies focusing on PC in patients with HFmrEF. The results of previous clinical studies on PC status in HFmrEF patients are sometimes difficult to interpret, since the enrolled “HFmrEF” patients in previous studies are in fact “HFpEF” or “HFrEF” patients per current definition. Skorodumova *et al*. [48] explored PC status in ADHF patients with HFmrEF through LUS. They found that after the remission of ADHF, pulmonary interstitial congestion was still dominant (the distance between B-lines was 7 mm), there was a small amount of pulmonary edema (the distance between B-lines was 3 mm), and the number of B-lines was related to the simultaneous detection of N-terminal pro-B-type natriuretic peptide (NT-proBNP) level and readmission. Obviously, more clinical studies are needed to explore the PC situation and change post various HF treatments in ADHF patients with HFmrEF.

## 6. Pulmonary Congestion in Acute Decompensated Heart Failure Patients with Preserved Ejection Fraction

HFpEF is defined as: (1) patients with symptoms and signs of heart failure; (2) LVEF ≥50%; and (3) objective evidence of cardiac structural and/or functional abnormalities consistent with the presence of LV diastolic dysfunction/raised LV filling pressures, including raised natriuretic peptides [10]. The pathophysiology of HFpEF is heterogeneous, including diastolic dysfunction, inflammation and oxidative stress/endothelial dysfunction, chronotropic dysfunction and cardiac reserve dysfunction, pulmonary hypertension and abnormal ventricular arterial coupling [49]. In HFpEF patients, parameters describing left ventricular filling may be normal or only slightly impaired. Exercise is helpful to reveal diastolic abnormalities that cannot be seen under resting conditions [50].

The main feature of HFpEF patients is the increased cardiac filling pressure at rest and further increased during exercise, the symptoms of ADHF patients with HFpEF are thus mainly related to PC. Reddy *et al*. [51] found that interstitial lung water was increased in ADHF patients with HFpEF, even during low-intensity exercise. The acute development of PC in HFpEF patients was associated with increased pulmonary capillary hydrostatic pressure and systemic venous hypertension associated with impaired RV-PA coupling. Simonovic *et al*. [52] evaluated the risk factors of PC in patients with HFpEF during exercise. They found that the formation of B-lines in patients with HFpEF during exercise was related to the deterioration of diastolic function, that is, PC was related to further aggravated diastolic dysfunction in patients with HFpEF. Rueda-Camino *et al*. [53] used bedside lung ultrasound to evaluate the PC status of HFpEF patients before discharge, the study found that patients with B-lines >15 at discharge faced 2.5 times higher risk of rehospitalization for acute heart failure than patients with B-lines <15.

## 7. Effects of Pulmonary Congestion-Guided Treatment in Acute Decompensated Heart Failure Patients with Different Ejection Fraction

As a whole, there are relatively few studies on PC status among ADHF patients with HFpEF, HFmrEF, and HFrEF. Most clinical trials are either in non-ADHF patients or ADHF patients without ejection fraction stratification. It is still controversial whether there are differences in PC status in ADHF patients with different LVEF (Table 2, Ref. [54, 55, 56, 57, 58, 59, 60, 61]). Yang *et al*. [55] showed that B-lines measured by lung ultrasound were positively correlated with early transmittal velocity to tissue Doppler mitral annular early diastolic velocity ratio (E/e’) and NT-proBNP, but negatively correlated with LVEF in both the HFpEF and HFrEF patients. The correlation of B-lines with E/e’ and NT-proBNP was superior to LVEF, especially in the HFpEF group. It has also been shown that E/e’ is useful for estimating pulmonary capillary wedge pressure in ADHF patients with HFpEF, but not with HFrEF [62]. Van Aelst *et al*. [63] evaluated the congestion markers [brain natriuretic peptide (BNP), mid-regional pro-atrial natriuretic peptide (MR-proANP), soluble CD146 (sCD146), and inferior vena cava (IVC)] in ADHF patients with HFpEF and HFrEF and demonstrated similar hemodynamic congestion in these patients. In contrast, other studies evidenced worse pulmonary congestion in ADHF patients with HFrEF as compared to ADHF patients with HFpEF. A multicenter prospective study included 314 patients with ADHF. The results showed that HFrEF patients had more severe PC and intravascular congestion than HFpEF patients at admission. In terms of response to diuretic treatment, the two HF phenotypes also showed some differences [61]. ADHF patients with HFrEF experienced greater remission of intravascular congestion after diuretics, while ADHF patients with HFpEF showed greater remission of PC [61]. Similarly, other studies demonstrated that the degree of PC was higher in HFrEF patients than that in HFpEF patients [56, 57], which may be the reason why HFrEF patients are more prone to decompensation.

**Table 2.** **Pulmonary B-line correlation studies with different ejection fraction**.

Study	Cohort	Zone	Position	N	Follow-up	Conclusions
Coiro *et al*. (2017) [54]	Hospitalized patients with AHF	28	Supine position	HFrEF (N = 59) (EF ≤40%)	-	LUS can identify patients with high BNP, but cannot identify patients with elevated E/e’, and also shows a prognostic role independent of atrial fibrillation status, EF or quantification time; The optimal B-line threshold seems to vary according to the quantitative time during hospitalization.
				HFpEF (N = 51) (EF >40%)		
Yang *et al*. (2017) [55]	Hospitalized patients with ADHF	8	Supine position	HFrEF (N = 32) (EF <50%)	6 months	There was no difference in B-lines between HFrEF and HFpEF; It has a good correlation between B-lines and E/e’, especially in the HFpEF group.
				HFpEF (N = 50) (EF ≥50%)		
Palazzuoli *et al*. (2018) [56]	Hospitalized patients with AHF	8	Semirecumbent position	HFrEF (N = 95) (EF <50%)	6 months	Compared with HFpEF patients, HFrEF patients had more B-lines and congestion at admission and discharge.
				HFpEF (N = 67) (EF ≥50%)		
Mozzini *et al*. (2018) [57]	Hospitalized patients with AHF	-	Supine position	HFrEF (N = 35) (EF <40%)	-	LUS can accelerate the discharge time of HF patients, and the B-lines clearance time of HFrEF patients is longer than that of HFpEF and HFmrEF patients.
				HFmrEF (N = 35) (EF 40–49%)		
				HFpEF (N = 50) (EF ≥50%)		
Dwyer *et al*. (2018) [58]	Outpatients with HF	8	Supine position	HFrEF (N = 73) (EF <50%)	12 months	Patients with HFpEF and HFrEF had similar congestion spectrum.
				HFpEF (N = 46) (EF ≥50%)		
Ceriani *et al*. (2020) [59]	Hospitalized patients with ADHF	28	Semirecumbent position	HFrEF (N = 74) (EF <50%)	4 years	The ultrasound score before discharge in HFpEF group was significantly lower than that in HFrEF group. The assessment of PC by LUS at discharge is related to the long-term mortality of hospitalized patients with heart failure.
				HFpEF (N = 75) (EF ≥50%)		
Gargani *et al*. (2021) [60]	Hospitalized cardiac conditions patients with AHF and non-AHF	28	Supine position	HFrEF (N = 199) (EF <50%)	14.4 months	B-lines >30 indicates that the prognosis of patients with HFrEF and HFpEF is poor. And B-line has more significant prognostic value for patients with HFpEF.
				HFpEF (N = 97) (EF ≥50%)		
Cogliati *et al*. (2021) [61]	Hospitalized patients with ADHF	11	Semirecumbent position	HFrEF (N = 142) (EF ≤45%)	90 days	At admission, the degree of PC in HFrEF was stronger than that in HFpEF. And the rate of congestion relief in HFrEF patients was faster than that in HFpEF.
				HFpEF (N = 172) (EF >45%)		

Abbreviations: N, patients enrolled; ADHF, acute decompensated heart failure; HF, heart failure; AHF, acute heart failure; LUS, lung ultrasound; PC, pulmonary congestion; HFrEF, heart failure with reduced ejection fraction; HFmrEF, heart failure with mid-range ejection fraction; HFpEF, heart failure with preserved ejection fraction; E/e’, the ratio of early transmittal velocity to tissue Doppler mitral annular early diastolic velocity; BNP, pro-B-type natriuretic peptide.

There is scanty literature on PC status among ADHF patients with different LVEF, there are even fewer studies reporting the impact of various interventions on PC outcomes in ADHF patients with HFpEF, HFmrEF and HFrEF. EPICC Study is a randomized, multicenter, single-blind clinical trial [64]. The protocol aims to prove that LUS-guided therapy improves the 6-month prognosis of HF patients. It will reveal whether HFrEF and HFpEF would have the same response to LUS-guided therapy [64]. More researchers are needed to demonstrate the distribution of lung water and mechanism of PC in ADHF patients with HFpEF, HFmrEF, and HFrEF in order to test the effects of targeted therapy on PC remission in these patients.

**Fig. 1.**
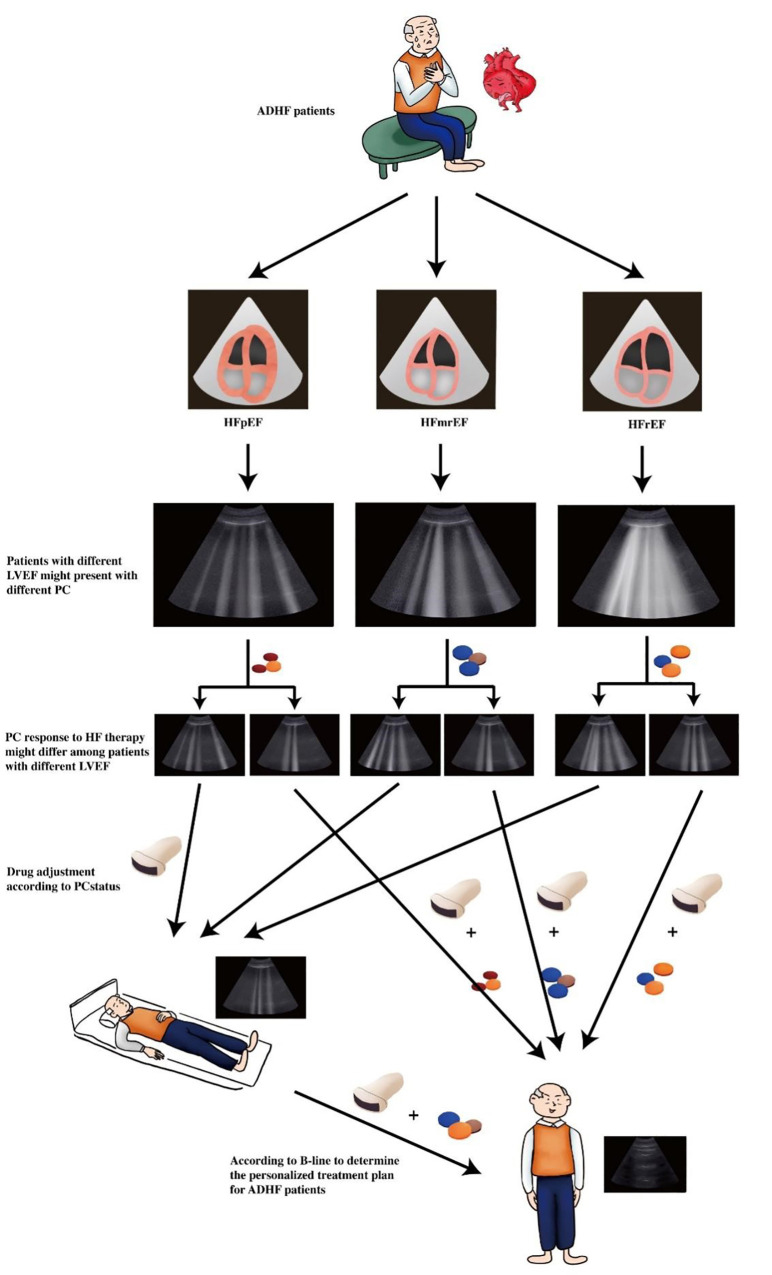
**Clinical value of lung ultrasound examination in ADHF patients with different left ventricular ejection fraction (LVEF)**. ADHF patients with different LVEF might have different pulmonary congestion (PC) status and response to therapy might also be different, repeated pulmonary congestion evaluation by lung ultrasound might be helpful for guiding drug adjustment aiming to attenuate pulmonary congestion in ADHF patients with different LVEF.

The treatment of ADHF mainly focuses on the remission of congestion. Persistent PC is a sign of poor prognosis in patients with ADHF [59]. The reduction in pulmonary venous congestion following the use of diuretics leads to a rapid improvement in dyspnea [65]. Although diuretics are important to alleviate congestion and symptoms in decompensated patients, randomized controlled trials have not demonstrated a prognostic benefit of these drugs up to now [66]. Angiotensin converting enzyme inhibitors (ACEI) can reduced cardiac filling pressure, mean arterial pressure, systemic vascular resistance and increase cardiac output in patients with congestive HF [66]. A clinical trial suggested that the benefits of high-dose mineralocorticoid receptor antagonists (MRA) therapy in acutely decompensated chronic heart failure (ADCHF) included lower natriuretic peptide levels, less congestion, better renal function, and less need for an intravenous diuretic [67]. Therefore, ACEI and MRA should also be considered as drugs to relieve PC regardless of EF (Fig. 1).

In patients with ADHF, the outcome of HFrEF was the worst in comparison with HFpEF and HFmrEF [11]. Kawase Y *et al*. [68] demonstrated that carperitide was associated with effective decongestion in the short term and satisfactory prognosis in the long term in AHF patients with moderate–severe PC, but carperitide was not associated with better clinical outcome in patients with no-mild pulmonary congestion. Selvaraj S *et al*. [69] proved that sacubitril/valsartan improved congestion more than enalapril. Reducing congestion in the outpatient setting is independently associated with improved quality of life and reduced cardiovascular events, including mortality. Sodium-glucose cotransporter-2 inhibitors (SGLT-2i) can help HFrEF patients by normalizing salt-water homeostasis to prevent clinical edema/congestion, so as to reduce hospitalization due to HF, improve functional status, quality, and duration of life in patients with HFrEF [70].

The characteristics of HFmrEF patients are between HFrEF and HFpEF. At present, there is no medication intervention study on the remission of PC among HFmrEF patients. In clinical practice, the treatment of HFmrEF patients is similar to that of HFrEF patients [71]. Drugs that alleviate PC in patients with HFrEF might also be effective for HFmrEF. However, this hypothesis should be validated with future clinical studies focusing on the effects of relevant therapy on PC status for HFmrEF patients in the setting of ADHF.

As for ADHF patients with HFpEF, literature on therapy and PC status is also limited. Park *et al*. [72] found that among patients with HFpEF (LVEF ≥50%), the mortality of patients with relatively lower LVEF was almost twice that of patients with stable LVEF. In HFpEF patients, the presence of orthopnea and PC predicted a higher risk of adverse cardiovascular events [73]. SGLT-2i have emerged as osmotic diuretics that may have utility in the treatment of HFpEF, by reducing renal glucose reabsorption and increasing urinary glucose excretion, SGLT-2i can thus relieve PC through natriuresis and diuresis [74, 75]. Málek F *et al*. [76] demonstrated that selective blockade of sympathetic signaling to the splanchnic circulation by surgical ablation of the right greatersplanchnic nerve (GSN) can reduce pulmonary capillary wedge pressure (PCWP) in HFpEF patients to improve quality of life and exercise capacity. Again, there is still no study describing the effects of relevant therapy on PC for HFpEF patients in the setting of ADHF.

## 8. Conclusions

LUS is a valuable tool to detect PC in ADHF patients and should be readily used in ADHF patients during hospitalization, before discharge and post discharge in the form of follow up or telemedicine. PC might increase in proportion to decreasing LVEF in ADHF patients. Timely detection and management of PC and regular PC monitoring by LUS post discharge might improve the outcome of ADHF patients. Importantly, response to diuretic and other heart failure medications might differ among ADHF patients with different LVEF. Obviously, more efforts are needed to monitor the responses and efficacy of applied treatment in ADHF patients with HFpEF, HFmrEF and HFrEF to find the best therapy options in terms of preventing the hospital readmission and improving the quality of life and outcome among ADHF patients with various LVEF.
